# Asymmetry in Erythroid-Myeloid Differentiation Switch and the Role of Timing in a Binary Cell-Fate Decision

**DOI:** 10.3389/fimmu.2013.00426

**Published:** 2013-12-05

**Authors:** Afnan Alagha, Alexey Zaikin

**Affiliations:** ^1^Nonlinear Analysis and Applied Mathematics Research Group (NAAM), Department of Mathematics, King Abdulaziz University, Jeddah, Saudi Arabia; ^2^Department of Mathematics and Institute for Women’s Health, University College London, London, UK

**Keywords:** GATA1-PU.1 switch, differentiation, immune cells, pluripotent cells

## Abstract

GATA1-PU.1 genetic switch is a paradigmatic genetic switch that governs the differentiation of progenitor cells into two different fates, erythroid and myeloid fates. In terms of dynamical model representation of these fates or lineages corresponds to stable attractor and choosing between the attractors. Small asymmetries and stochasticity intrinsically present in all genetic switches lead to the effect of delayed bifurcation which will change the differentiation result according to the timing of the process and affect the proportion of erythroid versus myeloid cells. We consider the differentiation bifurcation scenario in which there is a symmetry-breaking in the bifurcation diagrams as a result of asymmetry in external signaling. We show that the decision between two alternative cell fates in this structurally symmetric decision circuit can be biased depending on the speed at which the system is forced to go through the decision point. The parameter sweeping speed can also reduce the effect of asymmetry and produce symmetric choice between attractors, or convert the favorable attractor. This conversion may have important contributions to the immune system when the bias is in favor of the attractor which gives rise to non-immune cells.

## 1 Introduction

The importance of studying the immune system has attracted mathematicians and biologists to discover more of its features in recent years. One of the mechanisms is to study the genetic networks that control the lineage commitment of hematopoietic stem cells, which produce the full range of blood cells, including the immune cells (1). Many mathematical models have been used to study the differentiation of progenitor cell into erythroid and myeloid lineages based on the expression of lineage-specific transcription factors GATA1 and PU.1, respectively (2, 3). An important question arises in these models about the causes of bifurcation and symmetry-breaking and whether they occur in response to intrinsic cues or extrinsic signals. In fact, the integration of both intrinsic and extrinsic factors has received an extensive attention to elucidate the roles of external signals in cell-fate decision processes, and most importantly its relationship to the production of immune cells (3–6). Another important and interesting factor that can affect the decision of the cell is the speed of external signals or the speed of crossing the critical region (7–9). Remarkably, varying control parameter with time has been studied in many other systems. Ashwin et al. (10) have investigated how the rate of change of a parameter (or input) imposes significant changes in the climate system. It is found that rapid change may force the system to move away from a branch of attractors. This dependence on the rate was referred to as R-tipping. Another more recent study (11) has discovered how the stress response in bacteria is determined by the rate of environmental change. An increase in environmental stress leads to a single uniform pulse of alternative sigma factor *σ^B^* activation, a general stress response pathway, with amplitude depending on the rate at which the stress increased. It is found that faster stresses lead to larger and sharper activation of *σ^B^*, reflecting the fact that the activation process is rate-dependent. A question naturally arises how rate dependent signaling will affect the immune cell-fate selection via a differentiation of progenitor cells. We have studied these phenomena in the most paradigmatic switch responsible for the differentiation of immune cells, the GATA1-PU.1 switch. Moreover, we have considered how the shape of external signals may have an impact in decision-making process. The paper is structured as follows, we review the model of Huang et al. (2) and investigate, in addition to the symmetric scenario, the asymmetric scenario in two ways: (i) under the effect of asymmetric change of parameters; and (ii) under the effect of external signals, using two kinds of signals (see Materials and Methods). Furthermore, we will test the effect of parameter sweeping speed on the distribution of trajectories in the attractors of the dynamical system.

## 2 Materials and Methods

### 2.1 The GATA1-PU.1 gene regulatory circuit

**Figure 1:**
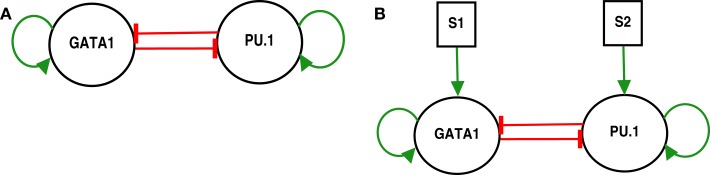
**GATA1-PU.1 genetic switch with and without external signals**. **(A)** The isolated switch consists of two transcription factors GATA1 and PU.1 that activate themselves while inhibit each other’s expression. **(B)** The exposure of the same switch in **(A)** to two external signals *S*_1_ and *S*_2_.

**Figure 2:**
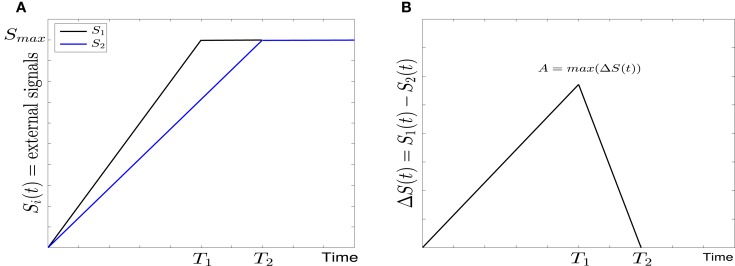
**Linear form of external signals in GATA1-PU.1 genetic switch**. **(A)** Two external signals *S*_1_ and *S*_2_ with different rising times but equal steady states at *S_max_* = 10. **(B)** The difference between the external signals with maximal asymmetry at *A*.

**Figure 3:**
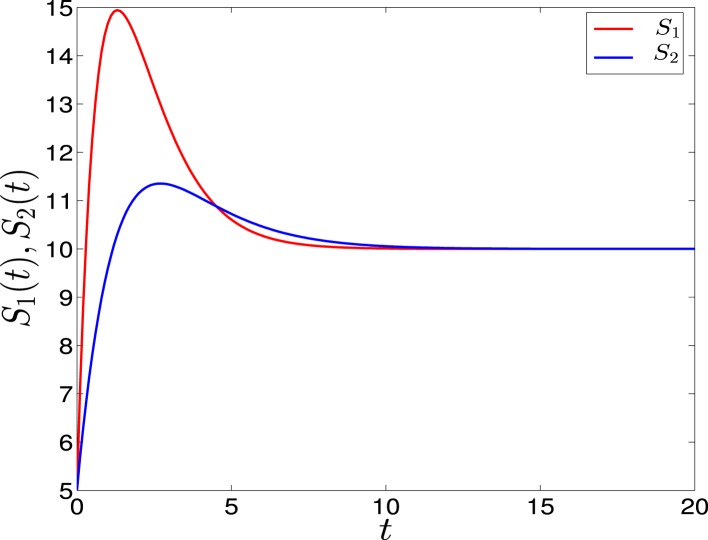
**Adaptation form of external signals in GATA1-PU.1 genetic switch**. The external signals *S*_1_ and *S*_2_ have different rising times but equal steady states at *v* = 10. Note that at the end of the signaling the system is structurally symmetric.

The model of the genetic switch responsible for differentiation contains mutual inhibition and is shown in (Figure 1A). The regulatory dynamics can be described by the following form (2):

(1) $$\begin{array}{ll} & \frac{dX_{1}}{dt}=\frac{a_{1}X_{1}^{n}}{r_{a_{1}}^{n}+X_{1}^{n}}+\frac{b_{1}r_{b_{1}}^{n}}{r_{b_{1}}^{n}+X_{2}^{n}}-k_{1}X_{1}+\sigma_{X_{1}}\xi_{X_{1}} \end{array}$$

(2) $$\begin{array}{ll} & \frac{dX_{2}}{dt}=\frac{a_{2}X_{2}^{n}}{r_{a_{2}}^{n}+X_{2}^{n}}+\frac{b_{2}r_{b_{2}}^{n}}{r_{b_{2}}^{n}+X_{1}^{n}}-k_{2}X_{2}+\sigma_{X_{2}}\xi_{X_{2}} \end{array}$$

where *X*_1_ and *X*_2_ are the concentrations of two transcription factors GATA1 and PU.1, respectively. These equations model the dynamics of self-activation and cross-inhibition with Hill functions (12). The parameters *a*_1_, *a*_2_ represent self-activation rates, the parameters *b*_1_, *b*_2_ are basal expression rates, *k*_1_, *k*_2_ are deactivation rates, the parameters *r*’s are thresholds at which the inflection point in the Hill function occurs, and *n* is the Hill coefficient. The first terms of equations (1) and (2) give the contribution from self-activation, while the second terms measure the effect of cross-inhibition on basal activation rates, and the third terms the degradation. To take account of intrinsic gene expression stochasticity, we consider the differential equations (1) and (2) in the Langevin form by adding multiplicative noise terms (the last ones) where $\xi_{X_{1}}$ and $\xi_{X_{2}}$ stand for a Gaussian noise and $\sigma_{X_{1,2}}$ depend on *X*_1,2_ as suggested in Ref. (13). These noise terms model the contribution of intrinsic noise which is unavoidable in biological systems. External cell signaling can be included in the model as follows

(3) $$\begin{array}{ll} & \frac{dX_{1}}{dt}=\frac{a_{1}S_{1}X_{1}^{n}}{r_{a_{1}}^{n}+X_{1}^{n}}+\frac{b_{1}r_{b_{1}}^{n}}{r_{b_{1}}^{n}+X_{2}^{n}}-k_{1}X_{1} \end{array}$$

(4) $$\begin{array}{ll} & \frac{dX_{2}}{dt}=\frac{a_{2}S_{2}X_{2}^{n}}{r_{a_{2}}^{n}+X_{2}^{n}}+\frac{b_{2}r_{b_{2}}^{n}}{r_{b_{2}}^{n}+X_{1}^{n}}-k_{2}X_{2} \end{array}$$

where *S*_1_ and *S*_2_ represent external signals to the genetic switch. Here, we are interested in two generic forms of signals:

- *Linear signals*: In this form (7) the external signals may have different rising times but they are equal in the steady state at *S_max_* = 10 (see Figure 2A). For the sake of simplicity we assume that *S*_1_ reaches to the steady state faster than *S*_2_, and thus the rising time *T*_1_ of *S*_1_ is smaller than the rising time *T*_2_ of *S*_2_. They both increase linearly with time according to
  (5) $$S_{1}(t)=\{\begin{array}{c} \begin{array}{ccc} \frac{S_{max}}{T_{1}}t & if & t\leq T_{1} \end{array}\\ \begin{array}{ccc} S_{max} & if & t>T_{1} \end{array} \end{array}$$
  (6) $$S_{2}(t)=\{\begin{array}{c} \begin{array}{ccc} \frac{S_{max}}{T_{2}}t & if & t\leq T_{2} \end{array}\\ \begin{array}{ccc} S_{max} & if & t>T_{2} \end{array} \end{array}$$
  The difference between *S*_1_ and *S*_2_ and the maximal difference *A* (Figure 2B) are defined as follows
  (7) $$\Delta S\left(t\right)=S_{1}\left(t\right)-S_{2}\left(t\right),\;A=max\left(\Delta S\left(t\right)\right)=S_{max}\left(1-\frac{T_{1}}{T_{2}}\right)$$
- *Adaptation form of signals*: As suggested in Ref. (14) to achieve biochemical adaptation the signals have transient growth stage where they reach to their maxima, and decay stage where they decay and saturate to their steady states (see Figure 3). As for the first form, *S*_1_ has a rising time, *θ*_1_, smaller than *S*_2_, *θ*_2_, and the value of saturation is 10. They have the following form
  (8) $$\begin{array}{ll} & S_{1}\left(t\right)=\frac{h_{1}}{\theta_{1}^{2}}te^{-\frac{t}{\theta_{1}}}+\frac{v}{1+e^{-t}} \end{array}$$
  (9) $$\begin{array}{ll} & S_{2}\left(t\right)=\frac{h_{2}}{\theta_{2}^{2}}te^{-\frac{t}{\theta_{2}}}+\frac{v}{1+e^{-t}} \end{array}$$
  where *h*_1_, *h*_2_ control the amplitude of signals, and *θ*_1_, *θ*_2_ are scale parameters. The second terms control the saturation of the signals to the value *v* = 10 (selected value). The maxima occur at *t_max_* = *θ*_1,2_. Consequently, we have chosen *θ* (*θ*_1_ or *θ*_2_) to be the value which determines the speed since as we increase *θ*, which represents the rising time, we increase the time at which the maximum occurs. In other words, we decrease the speed of the signal variation.

### 2.2 Testing of the parameter sweeping speed

To test the effect of speed, we compute the ratio *R* numerically using

(10) $$R=\frac{N_{u}}{N_{t}}$$

It represents the ratio between trajectories or cells which go to the top (or to the upper branch) of the bifurcation diagram, and trajectories that go to both upper and lower branches during simulation. Obviously, *R* = 1 if all cells choose the upper branch in the decision of their fate, *R* = 0.5 if the proportions of cells between two branches are equal, and *R* = 0 if all cells prefer the lower branch.

Heun’s method is used for solving the differential equations. In simulation of stochastic differential equations we have used Matlab, and all bifurcation diagrams and nullclines were generated in XPPAUT. δ(*t*) is an integration step size.

## 3 Results

### 3.1 GATA1-PU.1 genetic switch without external signals

This switch (Figure 1A) represents a paradigm for gene regulatory networks that govern the differentiation (2). It consists of two transcription factors GATA1 and PU.1 with self-stimulation and cross-inhibition. GATA1 is a master regulator of the erythroid lineage, and PU.1 is a master regulator of the myeloid lineage, and the two lineages arise from a common myeloid progenitor cell (1, 15).

#### 3.1.1 Bifurcation analysis for symmetric scenario

In the symmetric scenario, the parameters of the model are changed symmetrically with respect to *X*_1_ and *X*_2_. Hence, the rates of self-activation, cross-inhibition, deactivation, and thresholds are equal for both transcription factors (see Materials and Methods). Then, this scenario is divided into two parts depending on the kind of bifurcation which results in during a change of the parameters.

**Figure 4:**
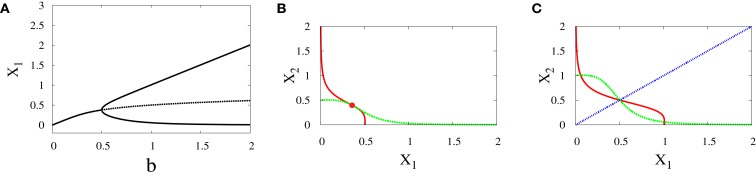
**Supercritical pitchfork bifurcation diagrams for symmetric scenario**. Bifurcation diagram **(A)** and nullclines at the beginning **(B)** and end **(C)** of the bifurcation. For all diagrams, *n* = 4, *r* = 0.5, *a*_1_ = *a*_2_ = 0.01, *k*_1_ = *k*_2_ = 1. The solid lines indicate stability, while dashed lines indicate unstable branches.

**Figure 5:**
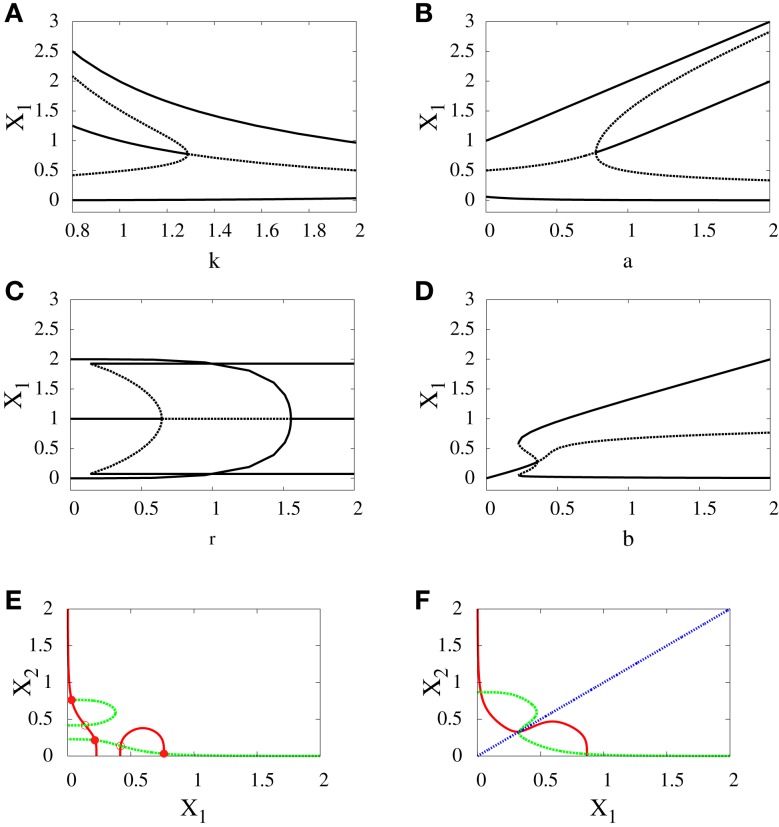
**Subcritical pitchfork bifurcation diagrams for symmetric scenario**. Bifurcation diagrams **(A–D)** and nullclines at the beginning **(E)** and end **(F)** of the bifurcation diagram **(D)**. For all *n* = 4. For **(A)** *a* = 1, *b* = 1, *r* = 0.5, **(B)** *b* = 1, *k* = 1, *r* = 0.5, **(C)** *a* = 1, *b* = 1, *k* = 1, **(D–F)** *a*_1_ = *a*_2_ = 1, *k*_1_ = *k*_2_ = 1.5. In **(C)** there is also supercritical pitchfork bifurcation.

- *Supercritical pitchfork bifurcation*: This type of bifurcation can occur when *b* is increased from 0.5 to 1 (Figure 4A), or when *r* is decreased from 1.8 to 1.2 (Figure 5C). In this kind of bifurcation, a transition occurs from monostability to bistability. The monostable state represents progenitor cell in undifferentiated state and has the ability to differentiate into two different fates. At this state, both transcription factors in the network are produced at approximately equal levels as it can be seen from the intersection point of nullclines in (Figure 4B). At the differentiation process, the progenitor cell is destabilized and two new attractors appear with equal basins of attraction (Figure 4C).
- *Subcritical pitchfork bifurcation*: This type of bifurcation occurs for many parameter changes. It can happen when *k* is changed from 1 to 1.5 (Figure 5A), when *a* is decreased from 1 to 0.5 (Figure 5B), when *b* is increased from 0.3 to 0.4 (Figure 5D), and when *r* is increased from 0.5 to 1 (Figure 5C). In this kind of bifurcation, a transition occurs from tristability to bistability (Figures 5E,F). In this situation, the progenitor cell (metastable state) coexists with the two fates, and the two transcription factors are expressed at equal or low levels. At the bifurcation process, it becomes unstable and makes discontinuous transition to either erythroid or myeloid fates with equal basins of attraction.

#### 3.1.2 Bifurcation analysis for asymmetric scenario

**Figure 6:**
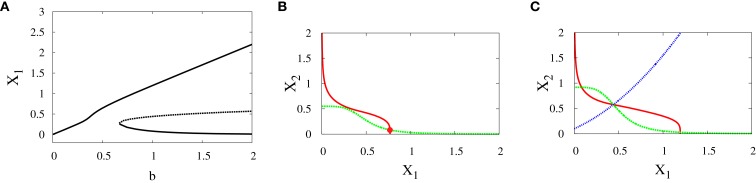
**Asymmetric scenario**. Supercritical pitchfork bifurcation diagrams. Bifurcation diagram **(A)** and nullclines at the beginning **(B)** and end **(C)** of the bifurcation. The parameters are *n* = 4, *r* = 0.5, *a*_1_ = 0.2, *a*_2_ = 0.01, *k*_1_ = 1, *k*_2_ = 1.1.

**Figure 7:**
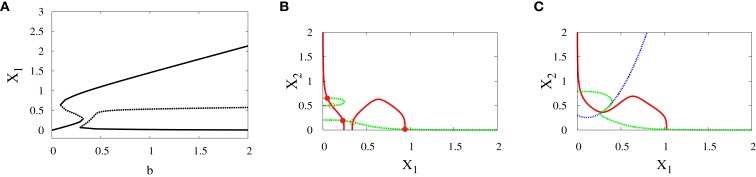
**Asymmetric scenario**. Subcritical pitchfork bifurcation diagrams. Bifurcation diagram **(A)** and nullclines at the beginning **(B)** and end **(C)** of the bifurcation. The parameters are *n* = 4, *r* = 0.5, *a*_1_ = 1.2, *a*_2_ = 1, *k*_1_ = 1.5, *k*_2_ = 1.6.

Here, the parameters of the model are changed asymmetrically with respect to *X*_1_ and *X*_2_. For example, we can increase one of the parameters and keep the other constant, or decrease one of the parameters and keep the other constant, or both. This asymmetric change will cause symmetry-breaking in the bifurcation diagrams and makes one of the attractors more favorable than the other. Similarly, we note two types of bifurcation:

- *Supercritical pitchfork bifurcation*: In order to get this kind of bifurcation with symmetry-breaking, we increase *a*_1_ and *k*_2_ (see Materials and Methods for their definitions) and as a result, *X*_1_ is increased. Then, the bifurcation occurs when *b* is changed from 0.6 to 1 (Figures 6A–C). Now, the uncommitted progenitor cell represented by monostability is not in the middle but at the point where *X*_1_ is higher. After bifurcation, the erythroid fate becomes dominant since it has a larger basin of attraction to the right of the separatrix (Figure 6C).
- *Subcritical pitchfork bifurcation*: The bifurcation occurs when *b* is varied from 0.3 to 0.4. This gives imperfect subcritical pitchfork bifurcation (Figure 7A). The change in system behavior from tristability to bistability is depicted in (Figures 7B,C). At the progenitor cell, both transcription factors have low levels but the progenitor cell is not exactly in the middle. After bifurcation, one of the attractors corresponding to erythroid lineage becomes dominant as a result of increasing self-activation of GATA1.

#### 3.1.3 Trajectories and the effect of parameter sweeping speed

**Figure 8:**
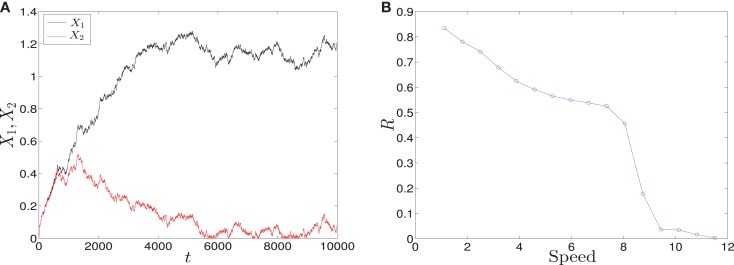
**Trajectories and parameter sweeping speed**. **(A)** Time evolution of *X*_1_ and *X*_2_ in the asymmetric supercritical pitchfork bifurcation. **(B)** The effect of increasing the speed of crossing the critical region on the distribution of trajectories in the attractors for 10000 iterations. As the speed is increased, the ratio *R* changes from 1 to 0. Hence, increasing the speed causes a large switch from the favorable attractor to the other one. Parameters are *a*_1_ = 0.2, *a*_2_ = 0.01, *k*_1_ = 1, *k*_2_ = 1.1, *n* = 4, *r* = 0.5. Also, in **(A)** *σ*^2^ = 0.01, **(B)** *σ*^2^ = 0.5.

To investigate the effect of the different speeds of the parameter sweeping we concentrate on the asymmetric supercritical pitchfork bifurcation, and similar results can be seen in the other kind of bifurcation. The graphical solutions of *X*_1_ and *X*_2_ after solving the differential equations (see equations (1) and (2) in Materials and Methods) are shown in (Figure 8A). As the time increases, the values of *X*_1_ increase and the values of *X*_2_ decrease. In fact, for small values of noise, this is the expected behavior from the dominance of the erythroid attractor.

To examine the effect of the speed with which the system crosses the critical region, we vary *b* linearly with time according to *b*(*t*) = *αt*, where *α* is the slope, and compute the ratio *R* (see Materials and Methods). The result is shown in (Figure 8B). For low speeds, the ratio *R* is high which means that most of the cells choose the erythroid lineage due to the produced asymmetry, and this lineage leads to and include red blood cells. On the other hand, as we increase the speed, this ratio tends to zero. Two conclusions follow from this behavior. First, large speeds reduce the effect of asymmetry gradually and convert the favorable attractor completely when the ratio tends to zero. Second, *R* = 0 means that the myeloid fate becomes more favorable by cells. The myeloid fate leads to the immune cells of the immune system (16).

### 3.2 GATA1-PU.1 genetic switch under external signaling

**Figure 9:**
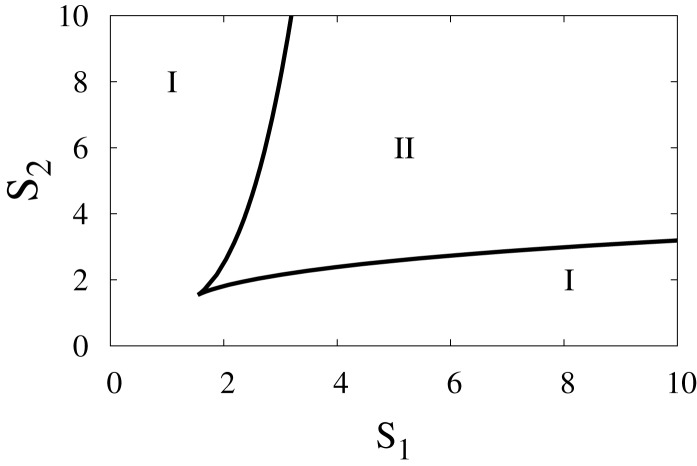
**Two-parameter bifurcation diagram**. The bifurcation in the parameter space (*S*_1_, *S*_2_), where *S*_1_ and *S*_2_ are external signals in the genetic switch. The borders separate between the regions of monostability *I* and the region of bistability *II*. Parameters are *a*_1_ = *a*_2_ = 0.05, *b*_1_ = *b*_2_ = 0.45, *r* = 0.5,*k*_1_ = *k*_2_ = 1.

To elucidate the effect of external signals on the dynamics of the switch, we consider external signals acting upon the switch (Figure 1B), see also equations (3) and (4). The external signals enhance the activation of *X*_1_ and *X*_2_. Figure 9 highlights the bifurcation in the parameter space (*S*_1_, *S*_2_) for the chosen set of parameters. The borders separate between the regions of monostability and the region of bistability, and this indicates to the existence of supercritical pitchfork bifurcation under the two following scenarios.

#### 3.2.1 Bifurcation analysis for symmetric scenario

**Figure 10:**
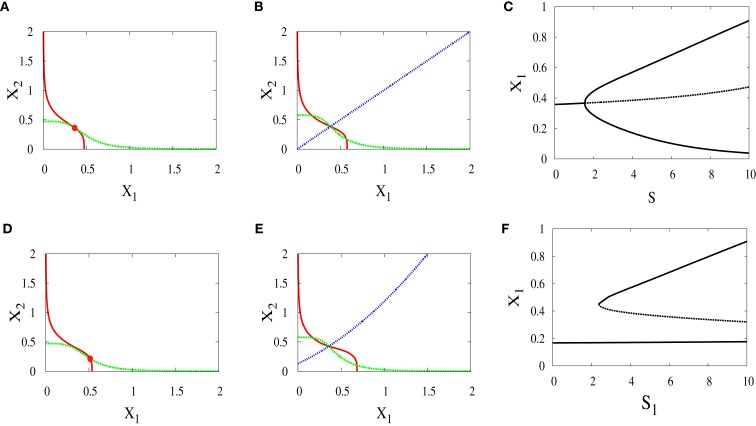
**Nullclines and bifurcation diagrams with symmetric and asymmetric external signals**. For all figures, *a*_1_ = *a*_2_ = 0.05, *b*_1_ = *b*_2_ = 0.45, *r* = 0.5, *k*_1_ = *k*_2_ = 1, *n* = 4. **(A)** Nullclines for *S*_1_ = *S*_2_ = 1 show one stable steady state, **(B)** nullclines for *S*_1_ = *S*_2_ = 4 show bistability, **(C)** near-symmetric supercritical pitchfork bifurcation, **(D)** nullclines for *S*_1_ = 3, *S*_2_ = 1 show one stable steady state shifted to the right, **(E)** nullclines for *S*_1_ = 6, *S*_2_ = 4 show bistability with larger basin of attraction to the right of the separatrix (almost diagonal line), **(F)** imperfect supercritical pitchfork bifurcation due to the asymmetry between the external signals.

Under this scenario, both signals *S*_1_ and *S*_2_ are equal. The nullclines in (Figures 10A,B) exhibit the bifurcation from monostability to bistability. This symmetry will give us near-symmetric bifurcation diagram (Figure 10C) with progenitor cell located in the middle and have equal probabilities to choose between erythroid (upper attractor) and myeloid (lower attractor) fates.

#### 3.2.2 Bifurcation analysis for asymmetric scenario

In contrary to the above scenario, now the signals have different parameters. As a result, the monostable state (Figure 10D) is at the point where *X*_1_ is higher since *S*_1_ which acts on *X*_1_ is larger. After bifurcation, the attractor at which *X*_1_ is high has a larger basin of attraction (Figure 10E). We can note in (Figure 10F) how this asymmetry produces symmetry-breaking in the bifurcation diagram and so the decision of the cell will be biased.

#### 3.2.3 Trajectories and speed-dependent cellular decision making

**Figure 11:**
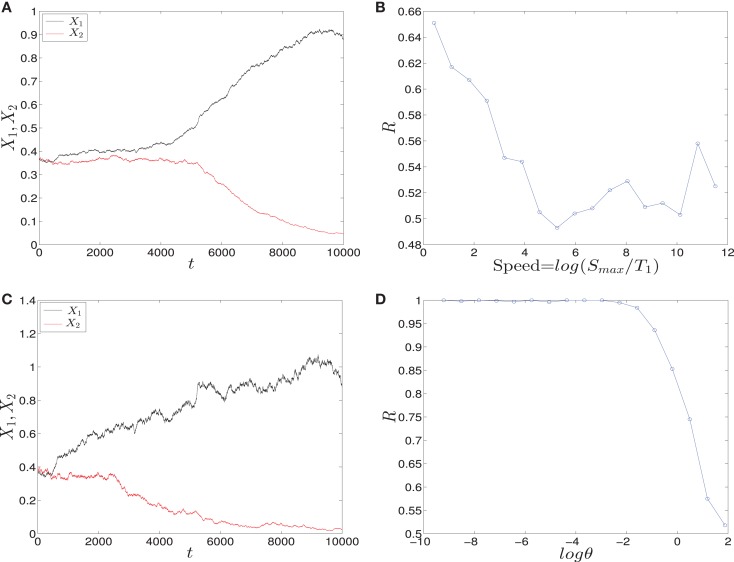
**Trajectories and effect of the external signaling speed**. In **(A,C)** the time evolution of *X*_1_ and *X*_2_ under the effect of linear and adaptation form of signals is shown, respectively. The values of *X*_1_ increase and the values of *X*_2_ decrease because *S*_1_ is chosen to be faster than *S*_2_. Hence, trajectories choose the attractor which has a larger value of *X*_1_. **(B)** The effect of increasing the speed of linear form of signals for 1000 iterations. As the speed is increased, the ratio *R* tends to 0.5. Thus, increasing the speed increases the symmetry in the switch. **(D)** The effect of speed with the adaptation form of signals. Decreasing the speed gives ratio *R* tending to 0.5. Surprisingly, now decreasing the speed increases the symmetry in the switch. Parameters in **(A)**, **(B)** are *A* = 2.5, *S_max_* = 10, **(C,D)** *h*_1_ = *h*_2_ = 10, *v* = 10, and for all we have *a*_1_ = *a*_2_ = 0.05, *b*_1_ = *b*_2_ = 0.45, *r* = 0.5, *k*_1_ = *k*_2_ = 1.

To study how signal asymmetry, noise, and decision making will result in the dependence of the parameter sweeping speed we have considered with two kinds of signals (See Materials and Methods):

- *Linear signals*: The signals are shown in (Figure 2A). The asymmetry between the two signals is transient and the symmetry is retained after some time (Figure 2B). The behavior of trajectories of *X*_1_ and *X*_2_ under the influence of this form of signals is shown in (Figure 11A). As the time increases, the values of *X*_1_ increase and the values of *X*_2_ decrease. Hence, trajectories of *X*_1_ and *X*_2_ choose the attractor at which *X*_1_ is higher since *S*_1_ is faster. Next, to test the effect of increasing the speed on choosing the attractors (stable steady states) of genetic switch in the presence of noise and transient asymmetry *A*, we vary *T*_1_ in $S_{1}\left(t\right)=\frac{S_{max}}{T_{1}}t$ with constant values of *A* and *S_max_*, and *T*_2_ will be changed according to the formula $T_{2}=\frac{S_{max}}{S_{max}-A}T_{1}$ (7). With increasing the speed (Figure 11B), the ratio *R* tends to 0.5. Thus, increasing the speed increases the symmetry between erythroid and myeloid lineages and reduce the effect of asymmetry which is produced by external signals.
- *Adaptation form of signals*: The signals take the non-linear form shown in (Figure 3) and as for the linear form, *S*_1_ is faster than *S*_2_. The trajectories in this form behave almost like the first form (Figure 11C). To study the effect of the speed, we vary *θ*_1_ and *θ*_2_ such that *θ*_1_ is smaller than *θ*_2_. Then, we compute the ratio *R* and the result is depicted in (Figure 11D). It shows ratio tending to 0.5 as *θ* is increased. But increasing *θ* decreases the speed, so, surprisingly, now we regain the symmetry in the switch by decreasing the speed of external signals.

## 4 Discussion

We have shown the importance of parameter sweeping speed when the gene regulatory circuit of immune cell differentiation is exposed to external factors that cause symmetry-breaking and make one of the attractors or fates more favorable than the other. In our study, symmetry-breaking is caused by three factors. The first factor is the asymmetric change of parameters which gives ratio tends to zero as the speed is increased (Figure 8B). This means we get large conversion from the favorite attractor, the erythroid lineage, to the myeloid lineage. The importance of this effect may appear in cases where the person has a problem with immunity due to the decrease in the production of immune cells, so even when there is a bias in the cell and this bias has the effect of choosing the attractor where GATA1 is upregulated, the cell can be forced to choose the attractor where PU.1 is upregulated by increasing the speed of crossing the critical region and so enhancing the production of immune cells. The second factor is linear form of signals (Figure 2A) and in this case we get ratio tends to 0.5 with increasing the speed (Figure 11B). This result may be important in situations that need symmetry between erythroid and myeloid cells, or when decreasing the probability of choosing the erythroid lineage is required. The third factor is represented by non-linear form of signals, i.e., signals describing biochemical adaptation (Figure 3). Here, decreasing the speed blinds the asymmetry and produce symmetry between the two lineages (Figure 11D). Taken together, the external signals, its shape, and its speed may have critical effects on choosing the attractors and affect the cell-fate determination.

Notably, we followed the model of Huang et al. (2) to study the differentiation into erythroid and myeloid fates. On the other hand, there is a scheme in Ref. (1, 17) that gives additional kinds of cells or lineages under each transcription factor. In this scheme, GATA1 is responsible for differentiation into erythroid or megakaryocyte cells, and PU.1 leads to either lymphoid lineage (B and T cells) which gives the *Adaptive Immune Cells*, or myeloid lineage (macrophages and granulocytes) that produces the *Innate Immune Cells*. So for this scheme, the importance of parameter sweeping speed is increased as the fate corresponding to high concentration of PU.1 is able to produce the different types of immune cells.

Of particular interest and agreement with our conclusions about the importance of external signals, Heuser et al. (6) have showed the crucial role of external signals in MN1 leukemia. They have investigated the requirement of FLT3 and c-Kit signals for MN1 leukemia. Overexpression of MN1 induces myeloid leukemia and blocks erythroid differentiation. FLT3 and c-Kit signaling direct MN1-expressing cells toward the myeloid lineage, so disruption of these signals may prevent leukemia. Interestingly, the disruption of these external signals doesn’t delay the disease latency but induces a switch from myeloid to erythroid lineage. Thus, the external signals can alter leukemia stem cell differentiation fates.

Many models have focused on the role of external signals in the differentiation process (3, 5) but they haven’t given any attention to the shape of signals or to the speed of these signals. Additionally, many works have made their studies limited to the symmetric scenario for the sake of simplicity (2, 18). But in this paper, we have studied the asymmetric scenarios and investigated the effect of external signaling speed on the system’s dynamics. As a prospect, it would be specially interesting to study the effect of speed on more complicated models and including other factors that may have a role in the differentiation process of hematopoietic stem cells, which can lead to better understanding of the immune system. Furthermore, an experimental evidence is needed to support the predictions from the mathematical models.

## Conflict of Interest Statement

The authors declare that the research was conducted in the absence of any commercial or financial relationships that could be construed as a potential conflict of interest.
